# The effect of refined nursing intervention on patients undergoing maintenance hemodialysis in the hemodialysis center during the COVID-19 epidemic

**DOI:** 10.1186/s12912-021-00584-5

**Published:** 2021-04-26

**Authors:** Qing-Lai Zhang, Shuo Wang, Yue Zhang, Fei Meng

**Affiliations:** Department of Blood Purification, Beijing Chaoyang Hospital Capital Medical University, No.8 of Gongti South Road, Chaoyang District, Beijing, China

**Keywords:** COVID-19, Maintenance hemodialysis, Refined nursing intervention, Intervention strategy, SCL-90

## Abstract

**Background:**

The outbreak of novel coronavirus pneumonia has exerted considerable psychological pressure on patients undergoing hemodialysis, resulting in unhealthy psychological emotions. Therefore, it is of great significance to carry out strict management and refined nursing intervention for patients undergoing maintenance hemodialysis during the prevention and control of novel coronavirus. This study aims to analyze and discuss the effect of clinical refined nursing intervention on patients undergoing maintenance hemodialysis during the coronavirus disease 2019 (COVID-19) epidemic.

**Methods:**

This was a prospective cohort study. In this study, we used the Symptom Checklist-90 (SCL-90) or the Chinese adult SCL-90 norm to conduct nursing interventions for patients undergoing maintenance hemodialysis to investigate the effect of clinical refined nursing intervention on patients undergoing maintenance hemodialysis during the COVID-19 epidemic.

**Results:**

The scores for all the factors of SCL-90 of patients undergoing maintenance hemodialysis were higher than those of the Chinese SCL-90, and patients with a single factor score ≥ 2 had a higher level of depression and anxiety, with extremely significant difference (*p* < 0.01). The depression and anxiety of the patients were reduced after the intervention, and there was a statistical difference. Among the 172 patients, the results of both nucleic acid tests were negative.

**Conclusion:**

During the COVID-19 epidemic, providing patients undergoing maintenance hemodialysis with refined nursing intervention can regulate negative emotions, reduce related complications, improve their quality of life, and improve the nurse–patient relationship.

**Supplementary Information:**

The online version contains supplementary material available at 10.1186/s12912-021-00584-5.

## Background

In early 2020, there was a sudden outbreak of novel coronavirus pneumonia in Wuhan that quickly spread across the country. Coronavirus disease 2019 is abbreviated as COVID-19. It is a new infectious disease caused by a new type of coronavirus that has the epidemiological characteristics of strong contagion, multiple routes of transmission, and wide spread [1–3]. COVID-19 is an infectious disease spread through respiratory droplets or contact such as coughing and sneezing. The disease is transmitted from person to person, with rapid onset, strong infectivity, and rapid changes in the course of the disease, and people are generally susceptible to it. Since patients undergoing maintenance hemodialysis have low self-immunity and poor resistance and most of them suffer from basic diseases, they are highly susceptible to the novel coronavirus [4]. Once patients are infected, the symptoms are severe and difficult to treat.

During the COVID-19 epidemic, we strengthened health education, enhanced publicity and guidance, and summarized, edited, and shared epidemic prevention and control knowledge as soon as was possible. The patients’ temperature was tested, health code was displayed, and nucleic acid test within 7 days was reported, and patients were asked about their travel trajectory on non-dialysis days. The body temperature of patients undergoing dialysis was tested four times on the day they came to the hospital for dialysis (before admission, before dialysis, after 1 h of dialysis, and after completion of dialysis). In clinical practice, we found that the outbreak of novel coronavirus pneumonia has exerted considerable psychological pressure on the patients undergoing hemodialysis, resulting in unhealthy psychological emotions [5] such as anxiety and depression. However, few studies have reported the effect of clinical refined nursing intervention on patients undergoing maintenance hemodialysis during the COVID-19 epidemic. Therefore, it is of great significance to carry out strict management and refined nursing intervention for patients undergoing maintenance hemodialysis during the prevention and control of novel coronavirus. This study aims to analyze and discuss the effect of clinical refined nursing intervention on patients undergoing maintenance hemodialysis during the COVID-19 epidemic to improve the negative emotions and the quality of life of these patients.

## Methods

### Design

This was a prospective cohort study. This study aims to analyze and discuss the effect of clinical refined nursing intervention on patients undergoing maintenance hemodialysis during the COVID-19 epidemic to improve their negative emotions and quality of life.

### Participants and setting

Using random sampling and the survey method, during the novel coronavirus epidemic, 172 of the center’s patients undergoing maintenance hemodialysis participated in this study. The random number table method was used to conduct a survey. The sample size was determined by 10–20 times the survey content. In this study, 10 factors were investigated in patients undergoing maintenance hemodialysis 10–20 times, which was 100–200 people. Finally, 172 patients participated in this study. The investigation was carried out between February 1, 2020 and July 1, 2020.

Inclusion criteria: dialysis treatment started more than 2 months ago; hemodialysis was conducted three times a week; there was no language communication and cognitive impairment, and patients could express their wishes accurately; patients had given informed consent. Exclusion criteria: patients with Alzheimer’s disease or other mental disabilities; patients with malignant tumors; patients with a history of mental illness; patients with limb paralysis; patients who refused to participate in this study.

### Intervention

The Symptom Checklist-90 (SCL-90) was used to conduct nursing interventions before, during, and after dialysis for patients undergoing maintenance hemodialysis, and the results were compared with the Chinese adult SCL-90 norm.

### Instruments

#### Symptom Checklist-90 (SCL-90)

The SCL-90 scale was first developed by L. R. Derogatis in 1954. It was revised in 1975 by the same author. SCL-90 is the most widely used psychological disease examination scale at present. It ranges from feeling, emotion, thinking, consciousness, and behavior to living habits, interpersonal relationships, diet, and sleep. It includes 9 subscales with 90 items, which are divided into 10 categories, namely 10 factors: somatization (reflecting subjective physical maladaptation), obsessive-compulsive symptoms, interpersonal sensitivity, depression, anxiety, hostility, paranoia, psychosis, and additional factors (reflecting sleep and appetite). Scoring criteria: a five-grade scoring system (from 1 to 5) was adopted ranging from none to serious. The score of each of the 90 items is added to obtain the total score. Total average score = total score / 90. Factor score = the total score of each item constituting a factor / the number of items constituting a factor. The score range is 1–5, the lowest is 1, and the highest is 5. The higher the score, the more obvious the symptoms [6] and the poorer the mental health. Internal consistency reliability (Cronbach’s coefficient) and split-half reliability were used to identify the homogeneity reliability of the scale. The Cronbach’s coefficient and split-half reliability of each scale were above 0.80.

SCL90 is one of the most famous mental health test scales in the world. It is the most widely used outpatient mental disorders and mental illness examination scale. SCL90 helps people to understand their mental health in 10 points. The Self-reporting Inventory, also known as the 90-item Symptom Inventory (SCL-90) or Hopkin’s Symptom Inventory (HSCL) was first developed by L. R. Derogatis in 1954. The revised version was developed in 1975 by the same author.

#### Refined nursing intervention methods

Refined nursing intervention is based on the principle of basic nursing, to maintain careful, painstaking, rigorous attitude in the nursing process, which is one of the modern high quality nursing mode. In this study, it included pre-dialysis, in-dialysis, and post-dialysis measures. The details of refined nursing intervention methods are listed below.

#### Nursing intervention before dialysis

##### Improve the nursing concept

To implement a refined nursing model for patients undergoing maintenance hemodialysis, it is necessary to start by improving the nursing concept of the nursing staff. The nursing staff adhere to the principle of precision and refinement in the process of nursing intervention, are careful in their daily work, and ensure no detail is missed. They need to have the right work attitude and provide patients with high-quality nursing services. The center cancels collective shifts and holds group work meetings twice a day. The head nurse presides over the meeting. Each group leader participates in the meeting. The head nurse conveys the department’s prevention and control requirements during the novel coronavirus epidemic and analyzes the spirit of related policies. The current difficulties faced by the team, urgent problems to be resolved, changes in critically ill patients’ condition, difficult vascular access, and other issues are outlined by the group leader during the meeting to jointly study solutions, and according to the dynamic changes of the epidemic situation, the problems in the management are analyzed and continuously improved. More than 90% of the problems reported by each group related to the psychological pressure exerted on patients undergoing dialysis by the epidemic, causing the patients to have negative psychological emotions. In response to this problem, the head nurse leads the nurses in conducting psychological counseling for patients undergoing dialysis, improving their negative psychological emotions, and resolving contradictions. To achieve this, it is necessary to do what should be done at once, nip the problem in the bud, effectively relieve the psychological pressure of patients, and ensure the normal progress of hemodialysis treatment.

##### Strengthen the training of nursing staff

If the professional level of nursing staff is not up to standard, it will hinder the implementation of the refined nursing model. Therefore, the department regularly strengthens the training of nursing staff. The training content is mainly based on theoretical knowledge and clinical nursing practice. The training involves enriching the nursing staff’s theoretical knowledge, improving their clinical practice ability, and improving their professional quality as a whole. During the epidemic, all the medical staff involved in blood purification participated in the COVID-19-related training sessions in Beijing and the hospital as required and met the standards.

##### Strengthen prevention and control measures

In the dialysis room, prevention and control measures were strengthened and prevention and control plans were formulated: isolation of the blood purification center area, closed management of the dialysis room, and strict regulation on two closures, namely closure of the front door of the dialysis room and closure of the treatment area. Family members are not allowed to enter the room, and, for patients who cannot take care of themselves, all the care work is undertaken by the responsible nurses and caregivers. Patients undergoing dialysis get on and off the machine at staggered peak, and issue special dialysis certificate, vehicle license, fixed family member, fixed vehicle, fixed shift, fixed area, strict division, allocated machines and time interval to avoid personnel gathering. Site cleaning is ensured before dialysis, and there is strict closed-loop terminal disinfection, strict and correct hand washing, correct wearing of masks, and a two-point one-line travel route. A pre-inspection team is set up. The inspection content is dynamically linked to the epidemic situation, and the inspection scope includes patients undergoing dialysis and their families. The order of investigation is test temperature, display health code, check result of nucleic acid test within 7 days, and ask the patients about their travel trajectory during non-dialysis days. The body temperature of patients undergoing dialysis should be tested four times on the day of dialysis (before admission, before dialysis, 1 h after the start of dialysis, and after completion of dialysis). If the body temperature is ≥37.3 °C during dialysis and accompanied by symptoms such as cough, dialysis is stopped for epidemiological investigation. In the waiting area, pictures and texts are used to make a one-meter interval sign, and cute little hedgehogs are pasted at the dividing points. The patients will experience a lift in mood when seeing the little hedgehogs, smile at each other, and make jokes such as “don’t prick yourself on that little hedgehog.” The humorous signs continuously remind patients not to gather or get together, and to adhere to the one-meter interval regulation during diagnosis and treatment. It is vital to effectively implement a series of prevention and control measures, ensure the safety of diagnosis and treatment in detail, leave no loose ends when implementing measures, leave no hidden dangers in the investigation, promote continuous improvement of work, be on strict guard, and ensure the safety of patients undergoing dialysis.

##### Health education

The outbreak of the novel coronavirus epidemic was sudden, fierce, and covered a wide area. It caused serious damage and had a great impact on the population, especially the vulnerable group of patients undergoing maintenance hemodialysis, who have suffered even more during this epidemic. The staff in the department have further strengthened health education; strengthened publicity and guidance during the epidemic; collected, edited, and shared the knowledge of epidemic prevention and control as soon as possible; and guided patients to treat the epidemic correctly and rationally without being nervous or afraid, advising them not to believe or spread rumors, be confident, actively cooperate with epidemic prevention and control work; and actively created an atmosphere of positive energy.

##### Nursing quality control

During the novel coronavirus epidemic, the responsibilities of nursing staff have been highlighted. During the implementation of refined nursing interventions, nursing staff must strictly control the quality of nursing, and each nursing link should be interconnected to ensure a complete and refined nursing process. Furthermore, nursing operation feedback information should be sorted out during the nursing process, and problems should be identified and resolved in time so that the quality of nursing is continuously improved.

#### Nursing intervention during dialysis

##### Strengthen education on epidemic prevention and control

Patients should be educated about COVID-19 prevention and obtain correct information about the epidemic, related prevention and control and popular science knowledge; patients’ awareness of COVID-19 should be increased, their fear and strangeness should be reduced, their psychological symptoms should be stabilized, and their confidence should be established to overcome the epidemic.

##### Psychological intervention

*Mental health management*: Since the outbreak of COVID-19, some patients undergoing dialysis have been under great psychological pressure and have different degrees of negative emotions, such as worry, anxiety, and depression [7]. The pressure comes from the lack of knowledge about COVID-19, the worry that dialysis treatment will be interrupted due to the epidemic, the worry that they or their caregivers will need to be isolated, and the feeling of helplessness. Feeling dull and depressed, wearing a mask throughout the dialysis process, they worry about hypoglycemia, thirst, and other forms of discomfort. To effectively relieve the psychological pressure of patients undergoing dialysis and ensure the normal progress of hemodialysis treatment, nursing staff should pay attention to the patients’ mental state, spirit, and emotion, actively communicate with patients, and adopt coping strategies. They should inform patients that they can bring some candy and straws for emergencies. In addition, they should use professional psychological care channels for patients undergoing dialysis, establish a platform for talk and interaction, face-to-face groups, WeChat groups, and video guidance to relieve stress, shoot anti-epidemic videos in dialysis rooms, improve immunity through exercise, and enhance anti-epidemic friendship through interaction. The video content and forms are diverse, such as playing the harmonica, poetry recitations, dancing with fans during dialysis, leg-raising exercises during dialysis, cheering for Wuhan, talent shows and competitions, and patients encouraging each other and strengthening friendships. Appropriate exercise can reduce the tension and anxiety of patients undergoing dialysis, eliminate pressure and anxiety in their lives, and make them feel more cared for. Moreover, it can enhance the nurse–patient relationship; improve the patients’ happiness index; improve their self-confidence so that they can feel strong enough to face life positively and optimistically, and achieve the goal of happy dialysis; and ultimately improve patients’ compliance with dialysis treatment and thereby improve their quality of life.

*Emotional support*: Patients undergoing maintenance hemodialysis usually receive treatment three times a week for 4 h each time. Most of the people they meet are medical staff and their wardmates. Nursing staff should adopt reasonable psychological intervention methods; actively care for patients and listen to patients’ complaints; encourage patients to talk about their worries, ideas, and treatment experiences; and encourage patients to share self-dialysis experiences. All these measures can effectively relieve the psychological pressure on patients. Emotional fragility and depression are among the most common psychological problems of patients undergoing dialysis [8]. Family members and dialysis partners are the main supporters of patients undergoing dialysis [9, 10]. Creating a good doctor–patient relationship and a harmonious patient–patient relationship can be beneficial. Nursing staff should provide emotional support to enhance patients’ ability to respond to COVID-19.

##### Nursing operation norms

Corresponding nursing operation procedures and norms should be formulated during the epidemic period, and nurses must strictly follow the rules and procedures in their daily work, ensure that every nursing link meets the standard requirements, and strengthen monitoring of key nursing links. Due to the different degrees of psychological problems of patients undergoing dialysis, during the treatment process, patients will have negative psychological effects due to excessive stress, which will affect their normal dialysis treatment. To effectively alleviate the psychological pressure of patients, nurses should “start from the heart.” If patients are asking for help when experiencing pain and helplessness, nurses are no longer indifferent and cold, but enthusiastically help and patiently comfort every patient with a smile, with love, enthusiasm, patience, sincerity, and responsibility. Nurses who are experienced and approved of by patients provide treatment and care, gain their trust and cooperation, create a good and harmonious nurse–patient relationship, and improve patient satisfaction, and thus win the honor for the department. After dialysis, the nursing staff must assist or accompany patients with limited mobility to leave the dialysis room until they are handed over to their family members to prevent adverse events.

#### Nursing intervention after dialysis

##### Implement effective surface and air ventilation disinfection

The dialysis room implements closed management and staggered on and off the machine, and clears the area completely for dialysis. Between the two shifts, 1000 mg/L chlorine disinfectant is used to wipe and disinfect the surface and ground, 500 mg/L chlorine disinfectant [11] is used to wipe and disinfect the high-frequency surface, detailed control is strengthened, high-frequency surface disinfection management of various surfaces is strengthened, and no loose ends are left. It is vital to ensure the air quality of the hemodialysis center, fully ventilate the departments, strengthen the ventilation between the two shifts of dialysis, and open the purification system throughout the whole process [12], perform terminal disinfection and keep records, and strictly control the closed-loop disinfection.

##### Strengthen prevention and control, prevent the spread of the epidemic, emphasize home self-management

It is crucial to improve prevention and control awareness and strengthen prevention and control during the epidemic. Patients go to the hospital for dialysis three times a week for 4 h each time. The other time is discharge time, so home self-management is particularly important. It is essential to fully implement the patient management system; for health education, it is necessary to comprehensively analyze, evaluate, plan, implement, and follow up to complete stratified, individualized, and systematic health guidance [13]; issue education brochures; strengthen the education of patients about prevention and treatment of COVID-19; and actively cooperate with the government, neighborhoods, and communities to perform prevention and control effectively.

Regarding monitoring and controlling home self-management, nursing staff should advise patients to pay attention to personal hygiene, keep the room clean and tidy, open windows for ventilation, maintain indoor air circulation, and keep warm during ventilation in winter to avoid catching colds. They should try to go out as little as possible, avoid contact with people with coughs and fevers, and avoid group activities such as parties to prevent the occurrence of cluster epidemics. If they must go out, they must wear a mask correctly and wash their hands before wearing a mask to maintain hand hygiene. It is necessary to distinguish the front and back of the mask, keep the dark side facing out, the metal strip on top. The mask should cover the mouth, nose, and chin, and the metal strip should be close to the bridge of the nose, so that the mask fits closely with the face. The key is to cover the mouth and nose completely. The mask should not be pulled down to the jaw or neck for the convenience of speaking and eating, and it should not be hung on the arm, which will contaminate the inner layer of the mask and lose its protective effect. The mask should be replaced in time if it gets wet or contaminated. When removing the mask, it is essential not to touch the outside of the mask with the hands. The elastic cords on both sides of the mask should be held with hands, the mask should be removed from both ears, the outside of the mask should be folded in and the inside out, and the mask should be thrown into the designated trash can. Hands must be washed frequently at home, and patients must not spit anywhere, pay attention to cough etiquette, cover their mouth and nose with a tissue or elbow sleeve when coughing or sneezing and after coughing or sneezing and when going home or receiving express delivery. They must use soap and running water or alcohol-based hand sanitizer to wash their hands correctly and develop good living and hygiene habits.

The patient’s body temperature and respiratory symptoms of novel coronavirus infection are monitored and reported in real time.

During the epidemic prevention and control period, patients undergoing dialysis should avoid changing the location of dialysis center; if they need to change it, it is recommended that they check with the original dialysis center after the epidemic is controlled, and then return. If the patient returns from other provinces, it is necessary to know whether there is contact history with confirmed or suspected infection cases, fever cases and their families, and there is no isolation at home. This should be handled in accordance with relevant isolation requirements, and appropriate protective measures should be taken.

Detailed records of fixed and hospitalized dialysis patients and their families’ home addresses, travel tracking and registration, and detailed records of the contact history of confirmed or suspected infected people, and home isolation or fever patients should be kept. The above information must not be concealed.

##### Diet instruction

Nutrients are the basic material for human growth and development. Good nutrition not only provides the human body with immunity against diseases including COVID-19, but also is the primary guarantee for promoting disease recovery. Therefore, dialysis patients should consume enough calories (30 ~ 35 kcal/kg/d) and high-quality protein (1.0 ~ 1.2 g/kg/d, at least 50% of which comes from high-quality animal protein), and vegetarian food is not recommended. They should properly control their water intake, especially in the hot summer season, and pay more attention to water intake. To reduce thirst, patients should refrain from consuming alcohol and food that is too salty and avoid drinking strong tea and strong coffee. If patients are really thirsty, they can rinse their mouth with water and spit it out. It is important to remember that the weight gain between two dialysis sessions should not exceed 3–5% of the patient’s dry weight. Weight and blood pressure should be measured at fixed points every day, accurately recorded and compared to assess whether the body’s moisture is balanced. Patients should adhere to a low-salt, low-potassium, and low-phosphorus diet, try not to eat raw food, and use separate cutting boards and tableware for raw and cooked food. Food should be washed and cooked. Hands should be washed before meals. During the epidemic, it is necessary to strengthen dietary hygiene and avoid eating during the night or eating unclean food, which may cause gastroenteritis and even peritonitis. It is vital not to touch, buy, and eat wild animals. Attention must be paid to the separation of raw and cooked food in the kitchen. Animal food should be cooked thoroughly. Family meals should be served individually, and public spoons and chopsticks should not be used to avoid mutual infection among family members.

##### Proper body-building exercise to improve immunity

Exercise has the effect of reducing negative emotions, enhancing physical fitness, and improving immunity. Exercise therapy uses the concept of sports medicine based on the patient’s functional situation. Active and passive exercises help patients improve their overall function and local function [14, 15]. Refined nursing intervention combined with exercise therapy measures for patients undergoing hemodialysis can enhance the physical fitness of the patients through scientific and appropriate exercises. In turn, it helps the patient develop a regular routine, maintains the body’s activity, produces effective stimulation to the nervous system, and puts the patient’s cerebral cortex in a relaxed state, thereby improving the patient’s sleep quality [16, 17] and helping the patient to regulate biological rhythms and improve daytime sleepiness, and effectively promotes the rationalization of sleep and rest [18]. Thus, it reduces the adverse effects of hemodialysis and diseases, and improves the physical functions and quality of life of patients. Exercise therapy for patients undergoing hemodialysis can help patients form good exercise habits. Exercise can improve patients’ nerve excitability, increase body happiness, enhance confidence in the disease treatment, relieve physical and mental stress, and relieve negative emotions such as depression and anxiety [19]. Nursing staff use their professional knowledge to help patients understand the benefits of exercise based on the patients’ condition and physical fitness, assist patients in formulating scientific and reasonable exercise plans and personalized exercise programs, and gradually and slowly increase exercise time and intensity through a step-by-step method. Along with a scientific nutrient supply, this helps patients maintain their energy levels, strengthen their physical functions, restore social functions, integrate more effectively into society, and increase participation in social activities, thereby improving patients’ physical and mental health and quality of life [20].

##### Improve and enrich patients’ spiritual and cultural lives

During the epidemic, patients undergoing maintenance hemodialysis must love life more, take an interest in current affairs, keep their mind open and at ease, overcome any anxieties and fears, arrange life in a reasonable and orderly manner, and cultivate more hobbies, such as painting, calligraphy, embroidery, and music. They should use interests and hobbies to inspire self-positive motivation, watch (listen to) more comedies and positively themed programs (books), and watch (listen to) less or not watch (listen to) programs with negative themes (books) to enrich their spiritual and cultural lives.

##### Popularize information technology and keep pace with the times

In recent years, with the improvement of the medical security system and medical conditions, the average life expectancy of human beings has increased, and the number of elderly patients with chronic renal failure has continued to increase. Therefore, the number of elderly patients undergoing maintenance hemodialysis is also increasing [21]. People are gradually realizing that improving the quality of care for elderly patients undergoing uremic maintenance hemodialysis is an important guarantee for improving the quality of life of such patients [22]. With the popularization of information technology, mobile phones have become an indispensable part of people’s daily lives, and people’s lifestyles have changed. However, elderly patients undergoing maintenance hemodialysis lack the mastery of new technologies and skills. Most of the patients’ mobile phones are phones specially designed for the elderly, and some patients do not even have a mobile phone and they do not know anything about the Internet and WeChat. To enhance patients’ ability to cope with epidemics and emergencies, nursing staff strengthen communication with patients, and carefully, patiently, and meticulously guide them to learn to use online platforms such as WeChat and QQ and online appointments, and patients actively learn new technologies and master new skills. Some patients have even bought a new smartphone for this purpose and actively cooperated with various measures to overcome obstacles and respond effectively to epidemics or emergencies.

### Data collection

In this study, 172 cases of patients undergoing maintenance hemodialysis in our hospital were selected. Between February 1, 2020 (before intervention) and July 1, 2020 (after intervention), the SCL-90 survey method was used for retrospective analysis. The “questionnaire star” was used to scan the QR code with WeChat to complete the questionnaire, and the questionnaires were distributed, explained, and collected by a designated person.

### Ethical considerations

This study was conducted in accordance with the Declaration of Helsinki and approved by the Ethics Committee of Chaoyang Hospital Affiliated to Capital Medical University.

### Statistical analysis

SPSS 21.0 software was used for the statistical analysis, and the measurement data were expressed by mean ± standard deviation. The count data was expressed as a frequency. W test was used to test the normality. T-test was used for comparison between the two groups that obey normal distribution, and nonparametric test was used for comparison between groups that did not obey normal distribution. *P*-value under 0.05 was considered statistically significant.

## Results

### General information

A total of 172 completed questionnaires were returned, and, therefore, the response rate was 100%, and no participants were lost to follow-up. Results of the survey conducted between February 1, 2020 and July 1, 2020 showed that the participants included 96 males and 76 females aged 26–84 years, with an average age of 53.13 ± 12.79 years who had been undergoing dialysis for more than 2 months. Regarding disease types, there were 46 cases of chronic glomerulonephritis, 56 high blood pressure kidney cases, 65 cases of diabetic nephropathy, and 5 cases of lupus nephritis. In terms of education level, there were 22 cases of junior high school and below, 123 cases of senior high school and technical secondary school, and 27 cases of junior college and above (see Table 1).

**Table 1:** The general characteristic

Item	Data
Male/Female	96/76
Age (Years)	53.13 ± 12.79
Chronic glomerulonephritis	46
High blood pressure kidney cases	56
Diabetic nephropathy	65
Lupus nephritis	5
Junior high school and below	22
Senior high school and technical Secondary school	123
Junior college and above.	27

### Comparison of SCL-90 scores of patients undergoing maintenance hemodialysis before and after refined nursing intervention

Before the psychological intervention for patients undergoing hemodialysis, the score of depression in the 10 factors of the SCL-90 was 2.38 ± 0.30, and the score of anxiety was 2.29 ± 0.27; after the intervention, the score of depression was 1.59 ± 0.35, and the score of anxiety was 1.57 ± 0.23 (t depression = 6.02, *p* < 0.01; t anxiety = 7.99, *p* < 0.01). The difference in depression and anxiety scores was statistically significant, whereas there were no statistical differences in other factors (*p* > 0.05). After the intervention, the patients’ depression and anxiety were alleviated (see Table 2). In addition, the results of both nucleic acid tests of all the patients were negative.

**Table 2:** Comparison of SCL-90 scores

factors	the outbreak began	epidemic situation	t	*p*
Somatization	1.36 ± 0.43	1.34 ± 0.37	0.03	> 0.05
Obsessive-compulsive disorder	1.43 ± 0.50	1.41 ± 0.46	0.48	> 0.05
Interpersonal sensitivity	1.37 ± 0.56	1.26 ± 0.45	0.01	> 0.05
Depression	1.51 ± 0.48	1.3 ± 0.4	2.41	< 0.05
Anxiety	1.52 ± 0.47	1.34 ± 0.52	4.87	< 0.05
Hostility	1.24 ± 0.44	1.20 ± 0.24	0.09	> 0.05
Phobic anxiety	1.26 ± 0.74	1.25 ± 0.37	0.63	> 0.05
Paranoid ideation	1.20 ± 0.36	1.17 ± 0.29	0.04	> 0.05
Psychoticism	1.28 ± 0.48	1.25 ± 0.56	0.12	> 0.05
Additional factors	1.52 ± 0.58	1.50 ± 0.61	0.56	> 0.05

## Discussion

According to the survey results, after the nursing intervention in the July epidemic stage, the patients’ anxiety and depression levels were significantly improved, with statistical differences. Results of both nucleic acid tests were negative, indicating that the patients did not have COVID-19 infection. Compared with other types of diseases, COVID-19 is more special and dangerous [23], and it poses a greater threat to the health of the patient and the surrounding population [24]. In addition to adopting a scientific and effective treatment plan, it should be supplemented with corresponding nursing intervention. The refined nursing intervention model should be applied to the clinical treatment of patients undergoing maintenance hemodialysis based on improving the nursing concepts of nursing staff, making nursing staff fully aware of the importance and value of the application of the refined nursing model, further strengthening nursing staff’s operating norms, improving nursing quality [25], fundamentally enriching nursing staff’s theoretical knowledge, and improving clinical practice capabilities to achieve refined nursing intervention for patients with maintenance hemodialysis and improve the overall quality of care. It is important to strengthen communication with patients and their families, so that they fully understand the COVID-19-related knowledge and precautions, control the spread of infectious diseases, and ensure the safety of patients and people around them [26].

Long-term maintenance hemodialysis treatment causes great economic and mental stress for patients, which can lead to negative emotions and reduced quality of life in patients [27, 28]. As one of the new models of modern nursing, the core of the refined nursing intervention model is humanistic care, which aims to provide patients with the highest-quality nursing services, effectively improve the quality of life of patients, and maximize their benefits [29]. This shows that applying the refined nursing intervention model for patients undergoing maintenance hemodialysis can help promote their health behavior compliance, correct their previous cognitive misunderstandings, improve their self-management ability and information practice awareness, improve their quality of life, reduce negative emotions, reduce the incidence of complications, and help maintain a good nurse–patient relationship, paving the way for the full implementation of nursing work [30]. In addition, the full companionship of the responsible nurse in the entire nursing work can promote the patients’ psychological satisfaction of being cared for and reduce the degree of unhealthy emotions such as tension. The emotional communication and careful care during the dialysis process can gain the trust of the patient, reduce their pain, and improve their psychological and physical comfort [31]. Patients’ dietary structure should be adjusted according to the characteristics and progress of their condition, and the intake of salt, potassium, phosphorus, and water should be limited, which help accelerate the prognosis of the disease and relieve the clinical symptoms [32, 33]. Therefore, through the above measures, the psychological and physical comfort of patients can be improved, and the two influence each other and cause each other to jointly improve the quality of life of patients, strengthen their belief in rehabilitation, and benefit the prognosis [34].

This study found that refined nursing intervention can regulate patients’ negative emotions, reduce related complications, improve their quality of life, and improve the nurse–patient relationship during the COVID-19 epidemic. However, there were several limitations in this study. First, this trial was not a randomized controlled trial. Second, this study was only a single-center trial, and the sample size was limited.

## Conclusion

During the COVID-19 epidemic, providing patients undergoing maintenance hemodialysis with refined nursing intervention can regulate their negative emotions, reduce related complications, improve their quality of life, and improve the nurse–patient relationship.

## Supplementary Information

**Additional file 1:.** Questionnaire.

## Data Availability

The datesets used or analyzed during the current study are available from the corresponding author on reasonable request.

## Abbreviations

SCL-90: Symptom checklist90
COVID-19: Coronavirus disease 2019
