# Supplementary material for: The effect of refined nursing intervention on patients undergoing maintenance hemodialysis in the hemodialysis center during the COVID-19 epidemic
Source: BMC Nurs. 2021 Apr 26;20:66. doi: 10.1186/s12912-021-00584-5 (PMC8072091; doi:10.1186/s12912-021-00584-5)
Supplement: Supplementary file 1 — Additional file 1:. Questionnaire. [file 12912_2021_584_MOESM1_ESM.docx]

**Dear patient:**

**Hello, due to the sudden emergence of the COVID-19, it has brought different levels of psychological impact to people, and dialysis patients are no exception. The purpose of this assessment: whether nurses' psychological nursing interventions for dialysis patients are effective during the prevention and control of the COVID-19. We will keep the content you filled in confidential, please feel free to answer, thank you very much for your cooperation!**

**Part 1:** General Information

**Part 2**: Symptom Self-rating Scale SCL-90

**Instructions**: the following table lists the symptoms or problems that some people may have. Please read each item carefully, and then select an appropriate number to fill in the answer box according to the degree to which the sentence is consistent with your own actual situation (**the last week or now**):

| \| **Gender** \|  \| **Primary disease** \|  \| \| --- \| --- \| --- \| --- \| \| **Age** \|  \| **Dialysis time (each time)** \|  \| \| **Education level** \|  \| **Dialysis frequency (weekly)** \|  \| \| **Occupation (before dialysis)** \|  \| **Dialysis month age** \|  \|   1— No: Consciously having no such problem  2—Very mild, consciously having this symptom, but without actual effect on the subject, or the effect is slight  3—Moderate: Consciously having this symptom, and with a certain impact the subject  4—A bit severe: Consciously having this symptom, and with a considerable impact on the subject  5—Severe: Conscious that the frequency and intensity of the symptoms are very serious, and the impact on the subjects is serious | | | |
| --- | --- | --- | --- | --- | --- | --- | --- | --- | --- | --- | --- | --- | --- | --- | --- | --- | --- | --- | --- |
| Serial number | Problems | At the beginning of the epidemic | At present |
|  | Headache |  |  |
|  | Nervousness, not at ease |  |  |
|  | Unnecessary thoughts or words lingering in the mind |  |  |
|  | Dizziness or fainting |  |  |
|  | Decreased interest in the opposite sex |  |  |
|  | Rebuking others for completeness |  |  |
|  | Feeling that others can control your mind |  |  |
|  | Blaming others for causing trouble |  |  |
|  | Forgetfulness |  |  |
|  | Worrying about the neatness of your clothes and the correct manners |  |  |
|  | Easily annoyed and excited |  |  |
|  | Chest pain |  |  |
|  | Fear of empty places or streets |  |  |
|  | Feeling that your energy is down and activity slows down |  |  |
|  | Wanting to end your life |  |  |
|  | Hearing sounds that others can't hear |  |  |
|  | trembling |  |  |
|  | Feeling that most people cannot be trusted |  |  |
|  | Poor appetite |  |  |
|  | Crying easily |  |  |
|  | Feeling shy and uncomfortable when getting along with the opposite sex |  |  |
|  | Feeling deceived, caught in a trap or someone wants to catch you |  |  |
|  | Suddenly feeling scared for no reason |  |  |
|  | Losing your temper uncontrollably |  |  |
|  | Afraid to go out alone |  |  |
|  | Often blaming yourself |  |  |
|  | Back pain |  |  |
|  | Feeling difficult to complete a task |  |  |
|  | Feeling lonely |  |  |
|  | Feeling depressed |  |  |
|  | Worrying too much |  |  |
|  | Not interested in things |  |  |
|  | Feeling scared |  |  |
|  | Easy to be hurt |  |  |
|  | Others can know your private thoughts |  |  |
|  | Feeling that others don’t understand you and don’t sympathize with you |  |  |
|  | Feeling that people are unfriendly to you, don't like you |  |  |
|  | Doing things very slowly to make sure they are done correctly |  |  |
|  | Very quick heartbeat |  |  |
|  | Nausea or upset stomach |  |  |
|  | Feeling inferior to others |  |  |
|  | Muscle ache |  |  |
|  | Feeling that someone is watching you, talking about you |  |  |
|  | Feeling difficult to fall asleep |  |  |
|  | Checking things over and over again |  |  |
|  | Feeling difficult to make a decision |  |  |
|  | Feeling afraid of taking trams, buses, subways or trains |  |  |
|  | Feeling difficult to breathe |  |  |
|  | Feeling a bout of chills or fever |  |  |
|  | Avoiding certain things, occasions, or activities because of fear |  |  |
|  | Feeling that your mind is empty |  |  |
|  | Feeling numbness or tingling in the body |  |  |
|  | Feeling a blockage in your throat |  |  |
|  | Feeling hopeless |  |  |
|  | Can’t concentrate |  |  |
|  | Feeling weak in a part of the body |  |  |
|  | Feeling nervous or easily nervous |  |  |
|  | Feeling heavy hands or feet |  |  |
|  | Thinking of death |  |  |
|  | Eating too much |  |  |
|  | Feeling uncomfortable when others look at you or talk about you |  |  |
|  | Having some ideas that are not yours |  |  |
|  | Having the urge to hit or hurt others |  |  |
|  | Waking up too early |  |  |
|  | Must wash hands repeatedly and count repeatedly |  |  |
|  | Unsteady sleep |  |  |
|  | Having thoughts about breaking or destroying things |  |  |
|  | Having some ideas that others don’t have |  |  |
|  | Feeling nervous about others |  |  |
|  | Feeling uncomfortable in crowded places such as shops or movie theaters |  |  |
|  | Feeling that everything is difficult |  |  |
|  | Feeling waves of fear or panic |  |  |
|  | Feeling uncomfortable eating in public |  |  |
|  | Often arguing with people |  |  |
|  | Feeling very nervous when alone |  |  |
|  | Others do not make proper evaluations of what you have achieved |  |  |
|  | Feeling lonely even with others |  |  |
|  | Feeling restless |  |  |
|  | Feeling worthless |  |  |
|  | Feeling that something familiar becomes strange or doesn't seem to be true |  |  |
|  | Yelling or throwing something |  |  |
|  | Afraid of fainting in public |  |  |
|  | Feeling that others want to take advantage of you |  |  |
|  | Troubled by some thoughts about sex |  |  |
|  | Thinking that you should be punished for your fault |  |  |
|  | Feeling that things will be finished quickly |  |  |
|  | Feeling that you have serious problems with your body |  |  |
|  | Never felt close to anyone else |  |  |
|  | Feeling guilty |  |  |
|  | Feeling something wrong with your brain |  |  |
